# Effects of habitat modifications on the movement behavior of animals: the case study of Fish Aggregating Devices (FADs) and tropical tunas

**DOI:** 10.1186/s40462-020-00230-w

**Published:** 2020-11-10

**Authors:** Géraldine Pérez, Laurent Dagorn, Jean-Louis Deneubourg, Fabien Forget, John D. Filmalter, Kim Holland, David Itano, Shiham Adam, Riyaz Jauharee, Sunil P. Beeharry, Manuela Capello

**Affiliations:** 1 MARBEC, Univ Montpellier, CNRS, Ifremer, IRD, Sète, France; 2 Unit of Social Ecology, Université Libre de Bruxelles (ULB), Bruxelles, Belgium; 3 South African Institute for Aquatic Biodiversity, Grahamstown, South Africa; 4 Hawai’i Institute of Marine Biology at University of Hawai’i, Kane’ohe, Hawai’i USA; 5 Institute for Marine and Atmospheric Reserch at University of Hawaiʻi, Mānoa, Hawai’i USA; 6 International Pole and Line Foundation, 1 London Street, Reading, RG1 4QW UK; 7 Ministry of Ocean Economy, Marine Resources, Fisheries and Shipping, Port Louis, Mauritius

**Keywords:** Acoustic tagging, Associative behavior, Density of floating objects, Movement behavior, Tropical tuna

## Abstract

**Background:**

Aggregation sites represent important sources of environmental heterogeneity and can modify the movement behavior of animals. When these sites are artificially established through anthropogenic actions, the consequent alterations to animal movements may impact their ecology with potential implications for their fitness. Floating objects represent important sources of habitat heterogeneity for tropical tunas, beneath which these species naturally aggregate in large numbers. Man-made floating objects, called Fish Aggregating Devices (FAD), are used by fishers on a massive scale to facilitate fishing operations. In addition to the direct impacts that fishing with FADs has on tuna populations, assessing the effects of increasing the numbers of FADs on the ecology of tuna is key for generating sound management and conservation measures.

**Methods:**

This study investigates the effects of increasing numbers of FADs (aggregation sites) on the movements of tunas, through the comparison of electronic tagging data recorded from 146 individuals tunas (yellowfin tuna, *Thunnus albacares*, and skipjack tuna, *Katsuwonus pelamis*) tagged in three instrumented anchored FAD arrays (Mauritius, Oahu-Hawaii and Maldives), that differed according to their distances among neighboring FADs. The effect of increasing inter-FAD distances is studied considering a set of indices (residence times at FADs and absence (travel) times between two visits at FADs) and their trends.

**Results:**

When inter-FAD distances decrease, tuna visit more FADs (higher connectivity between FADs), spend less time travelling between FADs and more time associated with them. The trends observed for the absence (travel) times appear to be compatible with a random-search component in the movement behaviour of tunas. Conversely, FAD residence times showed opposite trends, which could be a result of social behavior and/or prey availability.

**Conclusion:**

Our results provide the first evidence of changes in tuna associative behavior for increasing FAD densities. More generally, they highlight the need for comparing animal movements in heterogeneous habitats in order to improve understanding of the impacts of anthropogenic habitat modifications on the ecology of wild animals.

**Supplementary Information:**

**Supplementary information** accompanies this paper at 10.1186/s40462-020-00230-w.

## Introduction

Animals evolve in a heterogeneous environment with which they interact through physiological and/or behavioral responses. As a result of habitat fragmentation, barrier effects, or resource changes, animal movements can change at multiple scales [35] and their migration routes can be altered [47]. Anthropogenic activities leading to modification in habitats can also result in changes in the way animals use and move through their environment. Currently, little is known about the behavioral plasticity of animals when environmental conditions change [45]. In the terrestrial habitat, waterholes represent important sources of habitat heterogeneity for several species [36, 48], as do floating objects for several fish species, in the pelagic habitat of the world’s oceans. Indeed, many pelagic fish, including tropical tunas (yellowfin tuna, *Thunnus albacares*, bigeye tuna, *T. obesus* and skipjack tuna, *Katsuwonus pelamis),* form large multi-specific aggregations around floating objects [6, 17]. Naturally occurring, floating objects include debris from trees and logs, which enter the ocean via river months or from the coast. Our understanding of the reasons that lead to such aggregations remains limited [6, 17]. Two main hypotheses have been formulated: the “meeting point” and the “indicator-log”. The “meeting point” hypothesis suggests that floating objects can be meeting points for tunas, facilitating the formation of larger schools [10, 17], which provide benefits to these social species. The “indicator-log” hypothesis asserts that tunas use floating objects as indicators of rich environments, as natural floating objects typically originate from highly productive areas (i.e., river mouth, mangroves) [21]. For centuries, fishers have used this associative behavior to their advantages [14], first targeting natural floating objects and then deploying man-made floating devices called Fish Aggregating Devices (FADs) in order to attract tunas and enhance the efficiency of their fishing effort [16, 34]. About, 40% of the world’s tropical tuna catch consists of fish associated with floating objects [8]. Coastal anchored FADs are generally deployed near shore to support artisanal tuna fisheries while drifting FADs are utilized offshore by industrial purse seine fisheries. The increasing use of FADs since the 1990’s has resulted in thousands of artificial floating objects being deployed in the open ocean on a regular basis, changing the pelagic habitat [1, 8, 9, 32]. Such modification has led to concerns regarding the impacts of FADs on the ecology of tunas [8, 9, 22, 31], as FADs could act as ecological traps (see [2, 44]). These studies highlighted the need for an improved understanding of the effects of increasing numbers of FADs on the behavior of tunas. The model developed by Sempo et al. [46] demonstrated that, for social fish species, increasing the number of floating objects could lead to fragmentation of fish schools. However, an evaluation of the effects of increasing numbers of FADs on tuna behaviour, through field-based experiments, is yet to be conducted. Both anchored and drifting FADs alter the natural environment, but anchored FADs are easier to access and study owing to their close proximity to the coast and fixed location. As such, understanding tuna behavior around anchored FADs can improve the general understanding of tunas behavior in relation to drifting floating objects [11]. Many studies have investigated the behavior of tuna at anchored FADs, using active tracking [4, 7, 23, 30, 43]⁠ or passive acoustic telemetry [12, 19, 37, 41, 42]⁠. FAD associations were observed to last from a few minutes to several weeks [12, 19, 37, 41, 42], clearly illustrating that FADs are able to retain tuna. Fitting survival curves to acoustic telemetry data showed that the behavioral process driving the time that tuna spend at FADs is independent of time [41, 42]. Using passive acoustic telemetry in the Oahu FAD array, Dagorn et al. [12] found that 17% of the observed travel times of tagged tuna between two FAD associations were short enough to be coherent with straight line movements. This was in line with results from several active tracking studies which observed tunas making “straight-line” movements between FADs [4, 7, 13, 23, 30]. Analysing these movements, Girard et al. [18] demonstrated that tunas are capable of oriented movements towards FADs from 4 to 17 km away, while for larger distances, tuna movements seemed to be driven by random-walk dynamics. From these studies, it is possible to hypothesize that tuna movements in a FAD array could be broken down into two components (or processes): (i) tuna move randomly as long as they do not perceive the presence of a FAD and (ii) they orient towards a FAD when they detect its presence, which could occur from 4 to 17 km from the FAD, follow the results of Girard et al. [18]. Finally, none of these studies have focused on the role of FAD densities on tuna behavior for a particular species and size class.

The main objective of our study was to investigate the effects of increasing the number of aggregation sites on the movement behavior of animals. For this purpose we used the FAD associative behavior of tunas as a case study, as this is currently a major conservation concern for fisheries management bodies ([25]; ISSF: [40])⁠. Our methodological approach was to compare the behavior of tuna (characterized through the detection of tagged individuals by receivers attached to FADs) among three different instrumented anchored FAD arrays in Mauritius and the Maldives in the Indian Ocean and Oahu-Hawaii in the Pacific Ocean, each with different distances between neighboring FADs (i.e. different FAD densities).

## Materials & methods

### FAD arrays

**Fig. 1:**
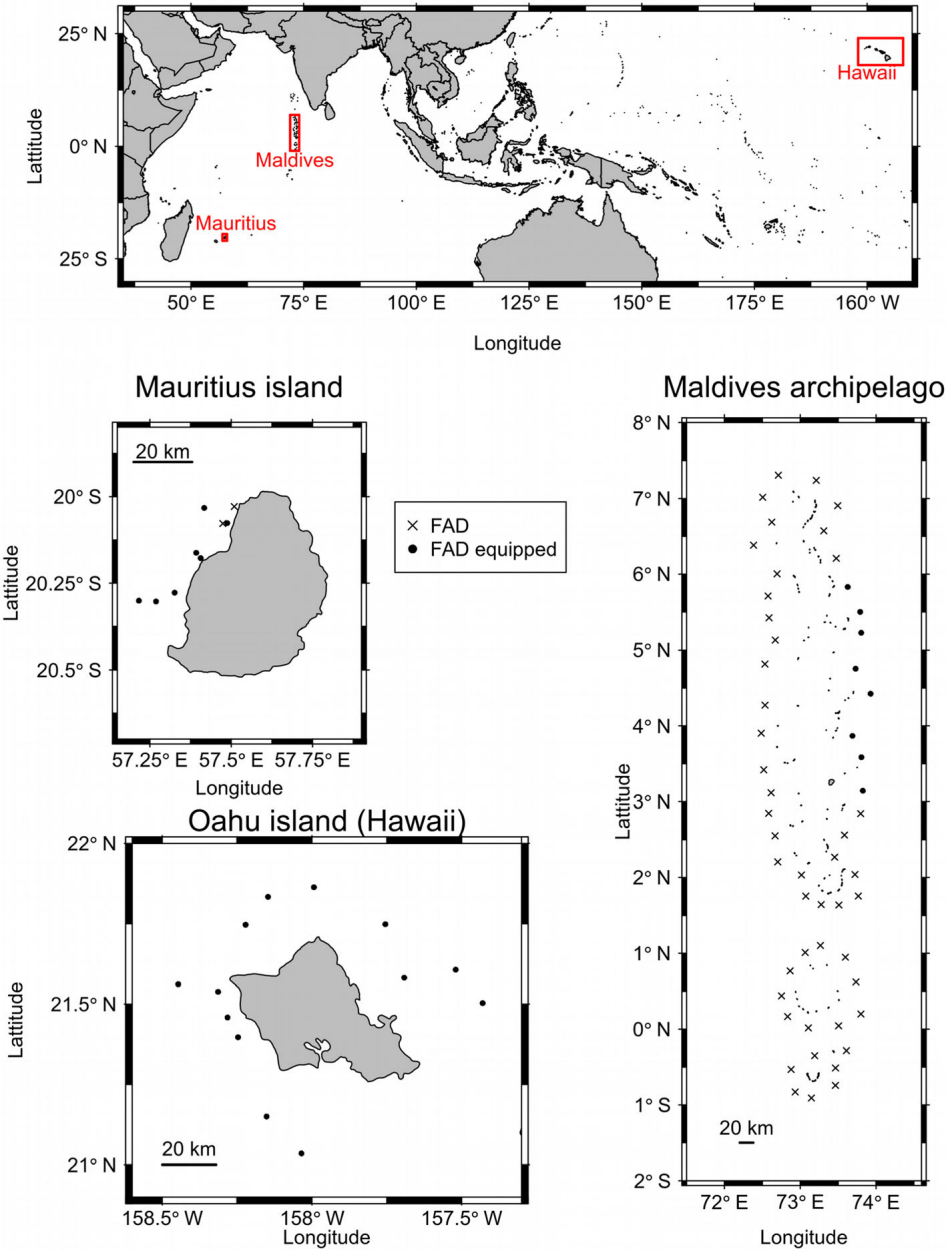
Location of the three anchored FAD arrays. The positions of anchored FADs are represented by a black dot when they were equipped with an acoustic receiver, and by a black cross when they did not have acoustic receivers

We used data collected from studies conducted around three anchored FAD arrays located around (i) the island of Mauritius [42] in the Western Indian Ocean (ii) the island of Oahu [41], within the Hawaiian archipelago, in the Central Pacific Ocean and (iii) the Maldivian archipelago [19] in the central Indian Ocean (Fig. 1). All FADs were moored in water between 1000 and 2500 m deep. FADs from the same array all had the same design, but designs differed slightly between arrays. The design of FADs, however, has never been identified to have an effect on the attractiveness of the floating objects, as tunas have been found around all types of floating object (see [17]). The Mauritian FAD array was composed of 9 FADs located on the western side of the island [42]. The Hawaiian array consisted of 13 FADs moored around the island of Oahu [41]. In the Maldives the array consisted of 54 FADs moored around the entire archipelago [19] (Table 1). The protocol for the three field studies was to exhaustively instrument all FADs of the same array (Hawaii) or sub-array (Mauritius, Maldives) (Fig. 1), in order to observe all movements between FADs located in the same area.

**Table 1:** Characteristics of the three instrumented FAD arrays

	Mauritius	Hawaii	Maldives
**No. FADs**	9	13	54
**No. FADs equipped**	7	13	8
**Nearest-neighbor distance (km)**	6 (3.0)	15 (4.1)	38 (7.0)
**Next-nearest neighbor distance (km)**	13 (2.7)	24 (9.3)	60 (13)

For the distances: mean values (standard deviation). No. FADs equipped denotes the number of FADs equipped with acoustic receivers

**Fig. 2:**
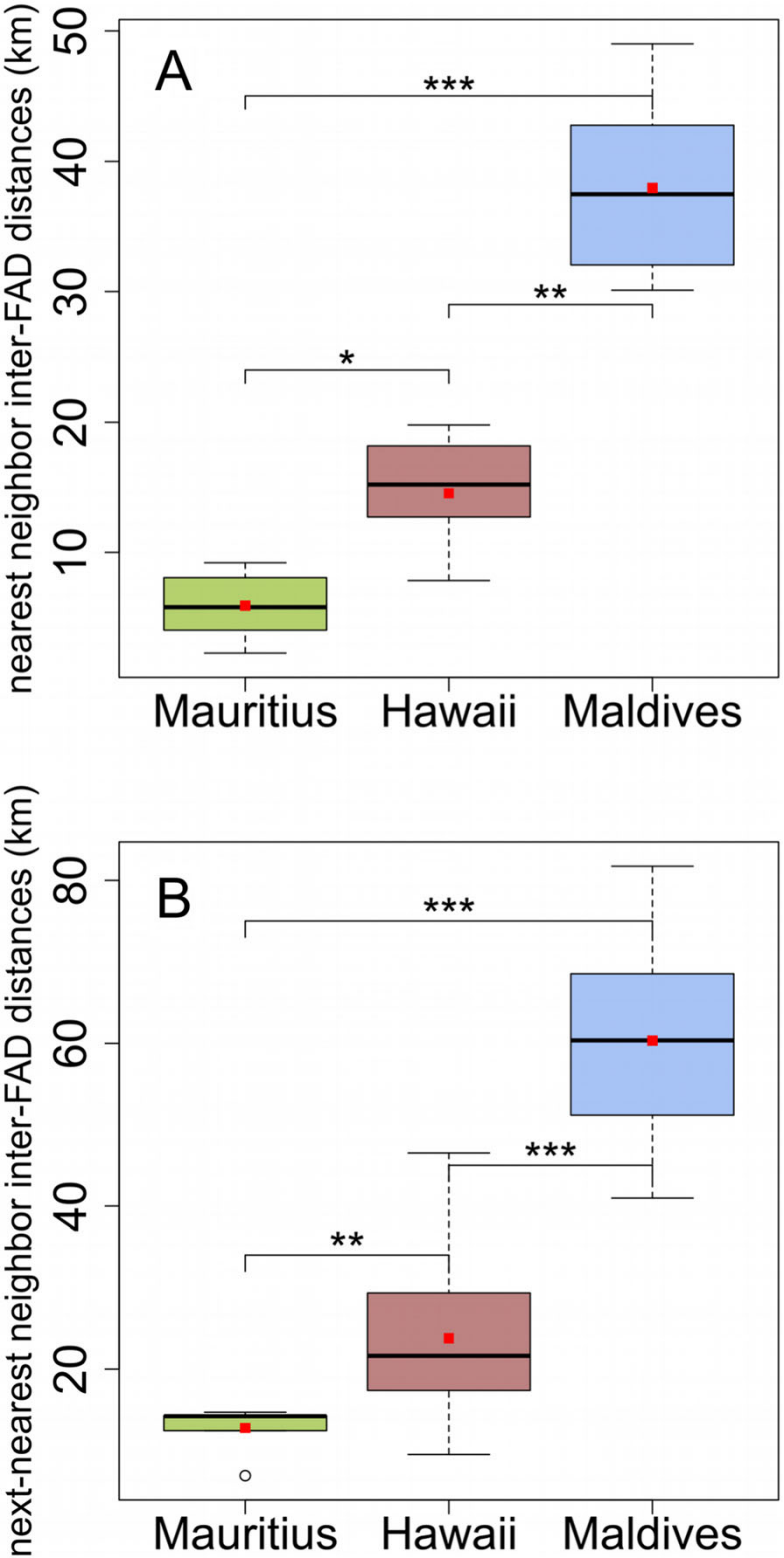
Inter-FAD distances of each study area. **a** nearest neighbor inter-FAD distances. **b** next-nearest neighbor inter-FAD distances. Dunn post hoc test with *p*-values adjusted by the Holm method: *** indicates *p* < 0.001, ** *p* < 0.01, and * *p* < 0.05

These three anchored FAD arrays were chosen as they each have different FAD densities, i.e., they offer different inter-FAD distances. Both the nearest-neighbor and next-nearest neighbor distances (Fig. 2a and b) were significantly different between the three instrumented FAD arrays (Dunn post hoc test, *p*-value< 0.05), see Fig. 2, and followed an increasing gradient from Mauritius – Hawaii – Maldives.

### Acoustic tagging database

During dedicated acoustic telemetry experiments, the three anchored FAD arrays were instrumented with acoustic receivers which detect the presence of tagged fish within a given detection range. Details of the telemetry experiments are described in Govinden et al. [19], Robert et al. [41] and Rodriguez-Tress et al. [42]. The available data sets collected during these experiments consisted of acoustic detections from 49, 92 and 52 tropical tunas tagged in the FAD arrays of Mauritius, Hawaii and the Maldives, respectively. The movements of tunas within the instrumented FAD arrays were monitored for between 38 and 50 days in Mauritius, from 3 months to 1 year in the Maldives and for more than 1 year in Hawaii.

The available detection data were grouped into cohorts according to species, size category and FAD array (Additional file 1). Additional file 2 provides the detailed numbers of tagged tuna within each category. To allow for the comparison of tuna behavior between different FAD arrays, only cohorts present in at least two FAD arrays, with at least 9 tagged individuals, were selected for the analysis (Additional file 2). This resulted in three species-size categories: yellowfin tuna of ~ 70 cm (YFT-70; fork length ranges: 60–80 cm), yellowfin tuna of ~ 50 cm (YFT-50; fork length ranges: 40–60 cm) and skipjack of ~ 50 cm (SKJ-50; fork length ranges: 40–60 cm). In total 146 tagged individuals were retained for the analysis. The YFT-70 category allowed for comparison between the Mauritian and Hawaiian FAD arrays with 14 and 56 individuals, respectively. The SKJ-50 category allowed for comparison between the Mauritian and the Maldivian FAD arrays with 15 and 22 individuals, respectively. The YFT-50 category was the only one where the comparison among the three FAD arrays was possible with 11, 9 and 19 tuna from Mauritius, Hawaii and the Maldives, respectively (Table 2).

**Table 2:** Indices for each species-size category and FAD array

	YFT-70		YFT-50			SKJ-50	
	Mauritius	Hawaii	Mauritius	Hawaii	Maldives	Mauritius	Maldives
**Ntuna**	14	56	11	9	19	15	22
**CRT (days)**	7.8 (8.8)	5.7 (11)	15 (15)	5.8 (7.5)	2.5 (4.4)	2.5 (4.4)	1.4 (2.6)
**CAT**_**return**_ **(days)**	2.6 (2.9)	9.8 (22)	1.7 (0.53)	3.9 (3.6)	–	3.0 (2.6)	–
**CAT**_**diff**_ **(days)**	0.79 (0.98)	3.9 (6.7)	0.64 (0.11)	11 (15)	–	2.8 (8.9)	–
**NCRT**	3.2 (2.5)	2.1 (2.3)	1.5 (0.52)	1.7 (0.87)	1 (0)	2.9 (2.2)	1 (0)
**NCAT**_**return**_	0.77 (1.0)	0.48 (1.1)	0.40 (0.70)	0.11 (0.33)	0 (0)	0.87 (1.5)	0 (0)
**NCAT**_**diff**_	1.5 (1.9)	0.7 (2.1)	0.20 (0.42)	0.67 (1.1)	0 (0)	1.1 (1.3)	0 (0)

Mean values (standard deviation). Number of tuna (Ntuna), number and mean duration (in days) per individual for each species-size category and FAD array. A CRT corresponds to the residence time at one FAD, a CAT_diff_ corresponds to the time between two consecutives visits at two different FADs and a CAT_return_ corresponds to the time between two visits to the same FAD with an absence longer than 24 h. The mean number of CRTs, CAT_return_ and CAT_diff_ were calculated for the first 38 days of tracking of each tuna, while the mean duration of CRT, CAT_return_ and CAT_diff_ were calculated over the entire observation time. The inter-FAD distances increase in order of Mauritius-Hawaii-Maldives

### Data analysis: associative and movement behavior

For each category, the time that tuna spent at (residence time), or away from (absence time) FADs was calculated. The residence times corresponded to the Continuous Residence Times (CRT) [5, 12, 37], which reflected the duration within which a tagged fish was continuously detected at the same FAD without day-scale (> 24 h) absences and without being detected at any other FAD in the array. The time that tuna spent away from FADs was defined as the Continuous Absence Times (CAT) [5, 19]. A CAT corresponded to the time interval between two consecutive CRTs. The times spent away from FADs were separated into two categories: (i) absences with returns to the same FAD of origin following periods longer than 24 h or, (ii) movements between different FADs in the array. Accordingly, the nature of consecutive CRTs (two consecutive visits to the same FAD or at different FADs) resulted in two different types of CATs: CAT_return_(s) and CAT_diff_(s).

The durations of CRT, CAT_return_ and CAT_diff_, were compared between FAD arrays for each species-size category, using the data recorded during the full study period for each array. When only two arrays were compared, a Mann-Whitney test [29] was performed. Where the three AFAD arrays were compared, a pairwise approach was used and a Kruskal-Wallis test [28] applied, followed by a Dunn post hoc test [15] with a *p*-value adjusted by the Holm method [24].

Finally, the number of return movements (NCAT_return_) and the number of movements between different FADs (NCAT_diff_), as well as the total number of residences times (NCRT) were also compared between FAD arrays for each species-size category. The total number of movements and residence times recorded over the full monitoring period within each array is shown in Additional file 3. To allow for comparison between study areas, each with different monitoring durations, only the number of residence times and movements recorded during the first 38 days of observation for all tuna tagged in all three FAD arrays were considered (Additional file 3). This duration is the shortest observation time recorded for a tuna in the three FAD arrays (it was recorded for a 50-cm YFT in the Mauritian array). Furthermore, NCAT_return_, NCAT_diff_ and NCRT were divided by the number of tuna tagged for each FAD array, to account for differences in tagging effort (Table 2 and Additonal file 5). The trends of these indices (CRT, CAT_return_, CAT_diff_, NCRT, NCAT_return_ and NCAT_diff_) were then compared with the output of a simple random-walk simulation, without any orientation nor retention component, for which all details are provided in Additional file 4.

All analyses were conducted using the R software (R Core Team 2018 version 3.4.4). Mann-Whitney tests were performed with the function “wilcox.test” (two-sample) in the “stat” package, Kruskal-Wallis tests were performed with the function “kruskal.test” in the “stat” package and Dunn tests were performed using the function “posthoc.kruskal.dunn.test” in the “PMCMR” package [39].

## Results

### Residence times

**Fig. 3:**
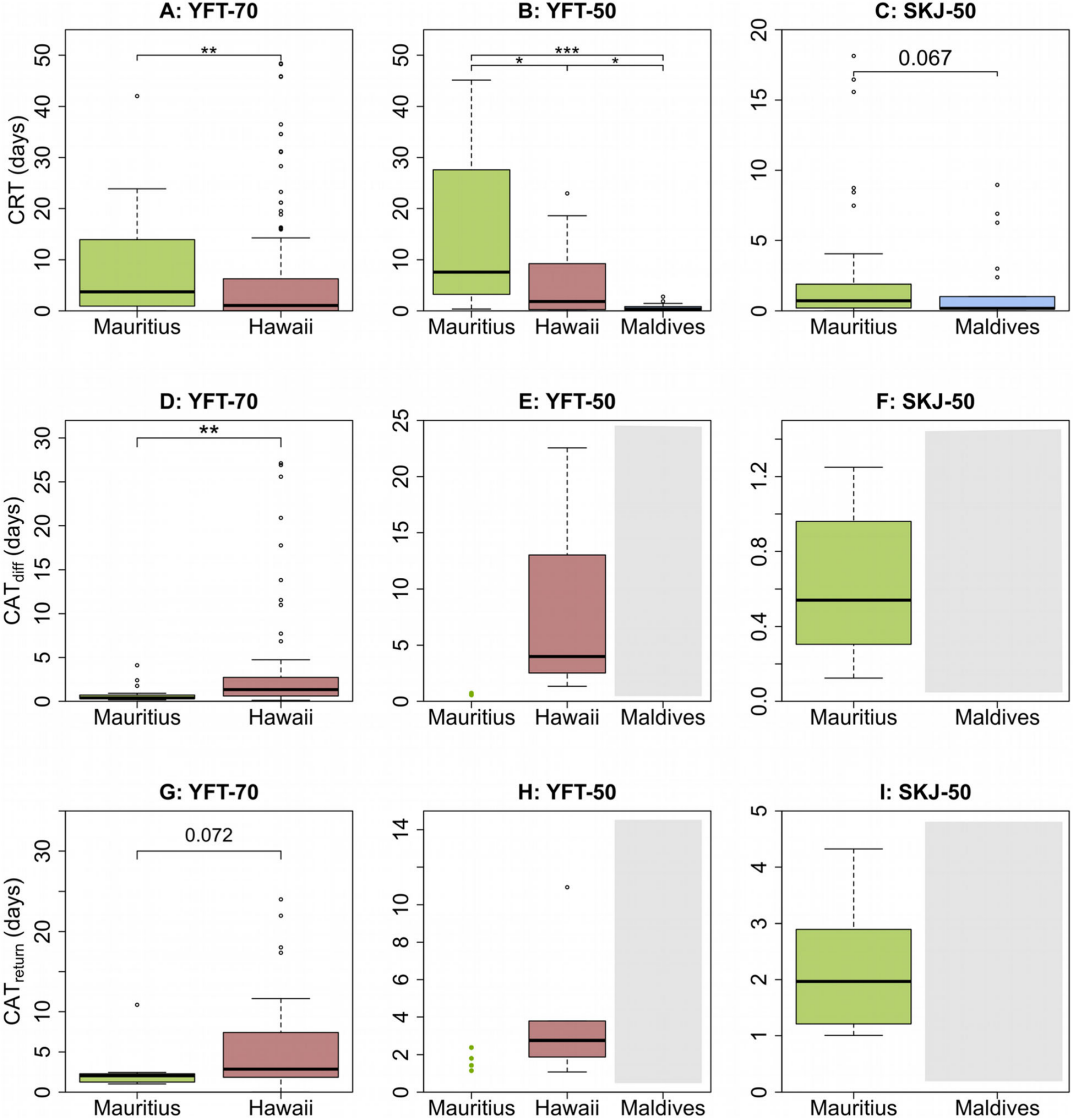
Duration of the CRT, CATdiff(s) and CATreturn(s) for the experimental data for each AFAD array and specie-size category. YFT-70 (**a**, **d** and **g**), YFT-50 (**b**, **e** and **h**) and SKJ-50 (**c**, **f** and **i**). Mann-Whitney test, except for YFT-50 where a Dunn post hoc test with *p*-values adjusted by the Holm method was performed: *** indicates *p* < 0.001, ** *p* < 0.01, and * *p* < 0.05

For the two YFT species-size categories, CRTs decreased significantly with increasing inter-FAD distances (Mann-Whitney test: *p*-value< 0.05 for YFT-70 and Dunn post hoc test *p*-value< 0.05 for YFT-50, Fig. 3a and b) and differences for SKJ were marginally significant (Mann-Whitney test: *p*-value = 0.067, Fig. 3c). In other words, the closer the FADs, the more time tuna spent in continuous association with the same FAD without interruption from visits to other FADs, or long excursions (> 24 h). The same significant decrease was observed for the number of CRTs for the three species-size categories (Mann-Whitney test: *p*-value< 0.05, Additional file 5), except for YFT-50 between Mauritius and Hawaii (Dunn post hoc test: *p*-value = 0.829, Additional file 5F). The average duration and number of CRTs per tagged individual for each species-size category and FAD array is shown in Table 2.

### Absence times

The mean numbers and durations of CAT_diff_ and CAT_return_ recorded for each species-size category and FAD array are shown in Table 2. For YFT-70, the durations of CAT_diff_ and CAT_return_ showed a significant increasing trend between Mauritius and Hawaii (Mann-Whitney: *p*-value< 0.05, Fig. 3d and g). This finding suggests that the greater the inter-FAD distances, the more time tuna spend away from FADs. Remarkably, no CAT (neither CAT_diff_ nor CAT_return_) was recorded in the Maldivian array (Table 2 and Fig. 3), which had the largest inter-FAD distances. This absence of CATs was significantly different from the number of CATs recorded in the other FAD arrays (see Additional file 5).

## Discussion

Habitat heterogeneity can play a crucial role in the movement ecology of marine animals. Relatively small structures can significantly impact animal movements either by inducing an attractive or retentive effect, or a combination of the two, resulting in animals spending a disproportionate amount of time in their proximity. Modifying the abundance of such structures can alter the movements of an animal, but very few studies have investigated the mechanisms by which such changes could result in alterations to animal behaviour [3, 27, 38]. Floating objects represent a key source of habitat heterogeneity for tropical tunas and other pelagic species, although the exact role played by these objects in the ontogeny or ecology of many species remains uncertain. While objects floating on the ocean’s surface were historically only of natural origins (e.g. logs), the number of artificial floating objects (e.g., FADs) has increased considerably in recent decades, surpassing the number of natural floating objects in some areas [9]. This addition of artificial objects to the pelagic environment is due to the significant fishing advantages that these devices afford. While concerns were raised more than two decades ago over the potential impacts these artificial objects could have on the ecology of tunas [31], no study has yet investigated, at a fine-scale, the effects of increasing the distances between floating objects on the movements of tunas. For the first time, the current study undertook a comparative analysis of the associative behavior of tunas with FADs, across multiple anchored FAD arrays with differing inter-FAD distances, in order to investigate how this factor could influence tuna movements.

### Absence time

**Fig. 4:**
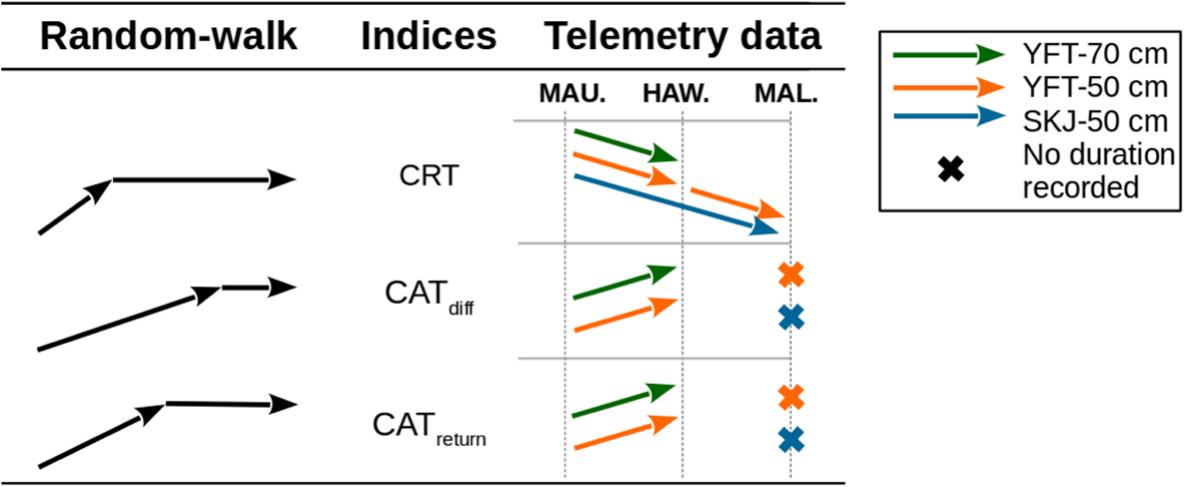
Summary of trends of three indices of movement behaviour. Trends are shown when inter-FAD distances increases. The first column shows the results of the random-walk simulations; the third column shows the results from the telemetry data analysis (MAU.: Mauritius, OAH.: Oahu, MAL.: Maldives)

The observed trends of CAT_diff_ and CAT_return_, which increased with inter-FAD distances, were similar to those found for the random walker (Fig. 4 and see Additional file 4). Knowing that CAT_diff_(s) and CAT_return_(s) correspond to periods when tagged fish are undetected, the significant absence of CAT_diff_ in the Maldives for all species and size categories may be interpreted as tuna performing excursions away from FADs longer than the observation time, as was observed in the random-walk simulations (see Additional file 4). The absence of CAT_return_(s) in the Maldivian FAD array may also be compatible with the random-walk trends observed in the simulations for finite observation times. Indeed, the simulated total number of CAT_return_(s) is expected to decrease at large inter-FAD distances and is likely to be very small for finite observation times (less than one CAT_return_ per simulated individual for inter-FAD distances larger than 30 km, see Additional file 4, Figure S2).

Despite the observed trends being compatible with those found for the simple random-walk model, the increased duration of movement between two different FADs (CAT_diff_) when inter-FAD distances increase may also be explained by tunas swimming in a straight line (oriented movement) from one FAD to another. With such oriented movements, the duration is proportional to the distance, and shorter than for movements with random components. Indeed, for a 50-cm individual with a speed of one body length per second, these durations would be 3.3 h, 8 h and 21 h for the Mauritian, Hawaiian and Maldivian array, respectively (mean neighboring inter-FAD distance of 6, 15 and 38 km, respectively, see Table 1). However, our analysis shows (Table 2) that durations of inter-FAD movements for 50-cm yellowfin tuna correspond in average to 0.6 (±0.11) days (±) and 11 (±15) days in the Mauritian and Hawaiian FAD arrays, respectively, while no CAT_diff_ were recorded in the Maldivian array. These values are larger than those expected for straight line movements between FADs. Previous studies of Girard et al. [18] found that tuna movements between FADs consist of two components: (i) oriented movements towards FADs at short distances and (ii) a random-walk dynamics at large distances. Adding an additional directed movement to FADs within a given radius to the random-walk behaviour will not change the trends in CAT_diff_ and CAT_return_ found for the simple random walk model. However, fitting a model that accounts for both this directed movement component and for the actual geometries of the FAD arrays (including the presence of the islands) on the field data is key for testing the hypothesis of a random-walk component in tuna movements more quantitatively.

### Residence time

Conversely, our results show that the evolution of CRT with increasing inter-FAD distances does not follow the trends found for the simple random-walk model (Fig. 4). This result outlines that the observed trends cannot be ascribed to the modified array of acoustic receivers but are a true signal of a change in tuna behavior (see Additional file 4). While possible effects of FAD densities in line with those predicted by the random-walk model cannot be excluded, the observed trends in CRT for increasing FAD distances indicate that the association time is driven by other processes. In this respect, the ‘meeting point’ [17] may explain a significant decrease of CRT for increasing inter-FAD distances. This hypothesis states that tunas could use FADs as meeting points to form larger schools. In simple terms, tunas could remain associated with FADs until the aggregation is large enough, then leave the FAD. When FADs are very numerous, under certain circumstances the local tuna population could be evenly distributed among FADs [46]. In such cases, the higher the FAD density, the smaller the sizes of the tuna aggregations under each FAD, and the longer they would stay at FADs in order to wait for their school to be large enough to leave (according to the ‘meeting point’). This effect may be amplified for increasing sizes of the tuna population in an area. As such, a large tuna population would imply larger aggregation sizes and thus shorter residence times. Considering that the Maldives has one of the largest anchored FAD fisheries in the world [19], significantly greater than in Mauritius and Hawaii, their (transient) local tuna population is certainly larger than those in the two other arrays. Consequently, under the meeting point hypothesis, the shorter recorded CRTs in the Maldivian array could be explained by the fastest formation of large tuna aggregations around FADs, due to both the low FAD density and the higher density of tuna within the array. This interpretation is valid when comparing the Mauritian and Maldivian arrays, as well as the Hawaiian and Maldivian arrays, but there is insufficient evidence to conclude that there is a difference in tuna density between the Mauritian and the Hawaiian arrays.

Ultimately, the trends in CRTs may also be explained by the availability of prey in the three anchored FAD arrays, as proposed in previous studies [37, 41]. Indeed, several studies demonstrated that tuna feed primarily on species that do not associate with FADs [20, 26, 33]. Since FADs do not have an important trophic function for tuna, individuals likely feed during their excursions away of FADs. The local availability of prey within the anchored FAD arrays may therefore affect the amount of time tuna spend at FADs. Consequently, longer residence times at FADs could correspond to higher prey abundance (and/or higher accessibility) in the Mauritian anchored FAD array than in the Hawaiian and Maldivian arrays.

## Conclusion and perspectives

The trends observed from tagging data for the absence times (travel time between two associations) appear to be consistent with the existence of a random search component in the behavior of tuna within a FAD array, in addition to the oriented component of the movements observed when tunas are a few kilometres from the FADs [18]. Conversely, the observed trends in FAD residence times appear to be driven by more complex processes involving, for instance, inter-individual interactions at the FADs, such as those suggested by the meeting point hypothesis, or due to prey availability. As our study is the first to focus on the role of FAD densities (inter-FAD distances) on tuna behavior for a particular species and size class, further studies on different FAD arrays would be necessary to strengthen our conclusions. However, we find the same trends for the three specie-size categories for the three arrays, knowing that one category compares the three arrays (YFT-50: Mauritius, Hawaii and the Maldives), and the two other compare two different arrays (YFT-70: Mauritius and Hawaii; SKJ-50: Mauritius and the Maldives).

Considering that when the FAD density increases, the connectivity between FADs increases (more FADs are visited), tuna spend shorter times un-associated and exhibit longer residence times, then the total time tunas spend in the FAD array will likely increase. Dedicated research is needed to quantify such change, for instance through a model of the behavior of tuna combining a random walk component with an oriented component.

The increase of the number of floating objects in the ocean would lead to increases in the time tuna spend at FADs, which would increase their vulnerability to fisheries. Such consequence can be extended to all species that associate with floating objects, including bycatch species such as dolphinfish (*Coryphaena hippurus*) or silky sharks (*Carcharhinus falciformis*), a vulnerable species. Consequently, understanding the effects of increasing the density of FADs on tuna behavior is clearly a major research objective for generating sound management and conservation measures to ensure the sustainability of this fishing practice.

In a broader context, this study can be extended to all wild animals that present an associative behavior with specific structures. We can cite for instance the importance of waterholes for terrestrial wild animals. The number and location of waterholes impact the space use by wild animals and their movement, as well as the viability of their populations, as for example if waterholes are inside or outside of protected areas [36, 48]. Changes of the number and/or location of waterholes may be caused by drought due to climate change [36] or caused by agriculture (livestock) [48]. In this respect, this study could open novel perspectives for comparing animal movements in a changing environment and improve knowledge on the impacts of global change on their ecology.

## Supplementary Information

**Additional file 1.** Number of tuna tagged per tuna size for each FAD arrays and tuna species.

**Additional file 2.** Number of tuna tagged in each FAD array per species and size category.

**Additional file 3.** Number of tuna, number of CRTs, CAT_return_(s) and CAT_diff_(s) for the full observation time and for only the first 38 days of observation.

**Additional file 4.** Random-walk model.

**Additional file 5. **Number of CRT (NCRT), CAT_diff_ (NCAT_diff_) and CAT_return_ (NCAT_return_) per individuals for each AFAD array and each specie-size category. YFT-70 (A, D and G), YFT-50 (B, E and H) and SKJ-50 (C, F and I). Mann-Whitney test, except for YFT-50 where a Dunn post hoc test with *p*-values adjusted by the Holm method was perfornmed: *** indicates *p* < 0.001, ** *p* < 0.01, and * *p* < 0.05. Binomial test: ### indicates *p* < 0.001, ## *p* < 0.01, and # *p* < 0.05.

## Data Availability

The datasets used and/or analyzed during the current study are available from the corresponding author on request.
