# Supplementary figures and images for: Effects of habitat modifications on the movement behavior of animals: the case study of Fish Aggregating Devices (FADs) and tropical tunas
Source: Mov Ecol. 2020 Nov 10;8:47. doi: 10.1186/s40462-020-00230-w (PMC7654007; doi:10.1186/s40462-020-00230-w)

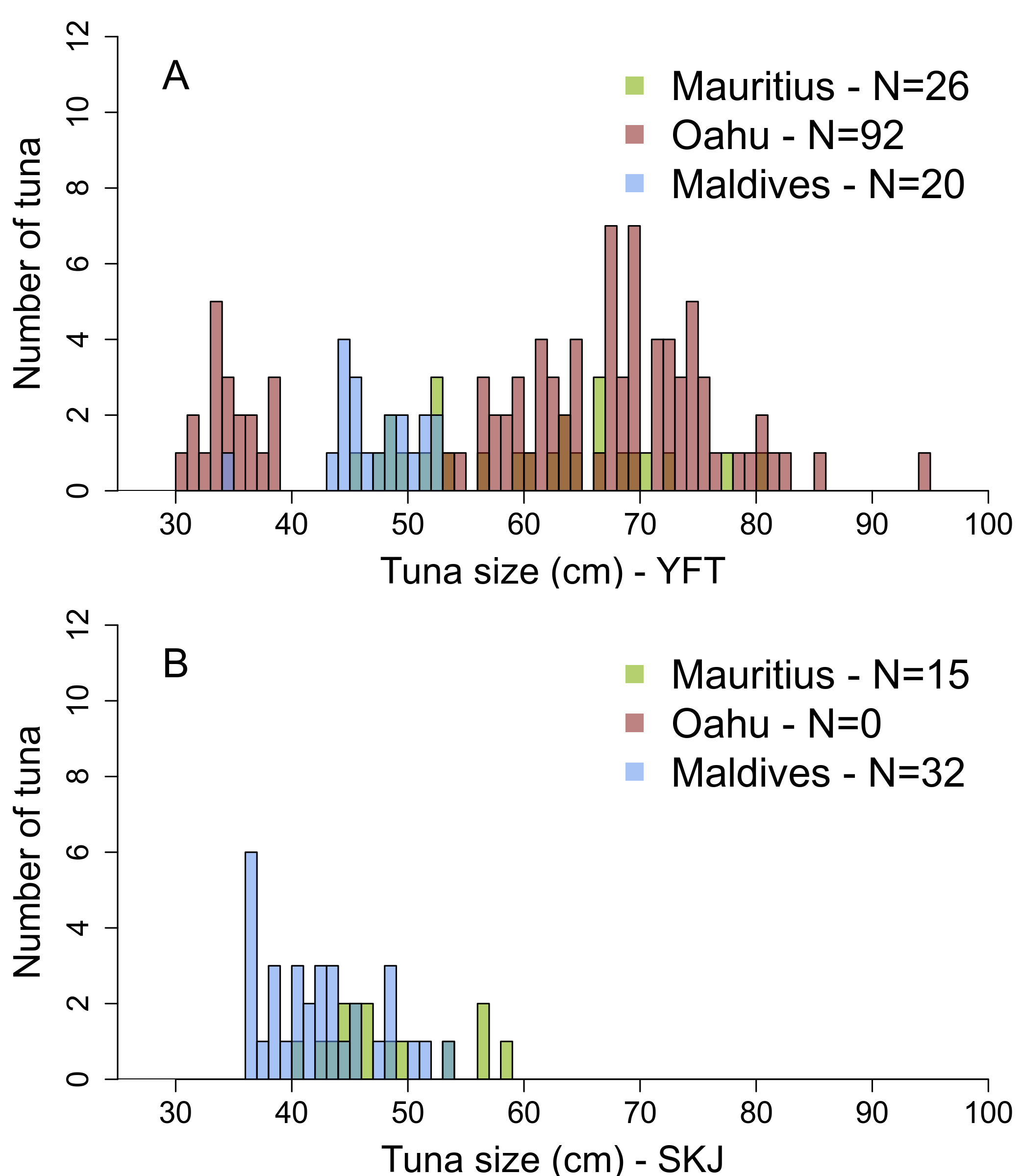

Supplement: Supplementary file 1 — Additional file 1. Number of tuna tagged per tuna size for each FAD arrays and tuna species. [file 40462_2020_230_MOESM1_ESM.png]

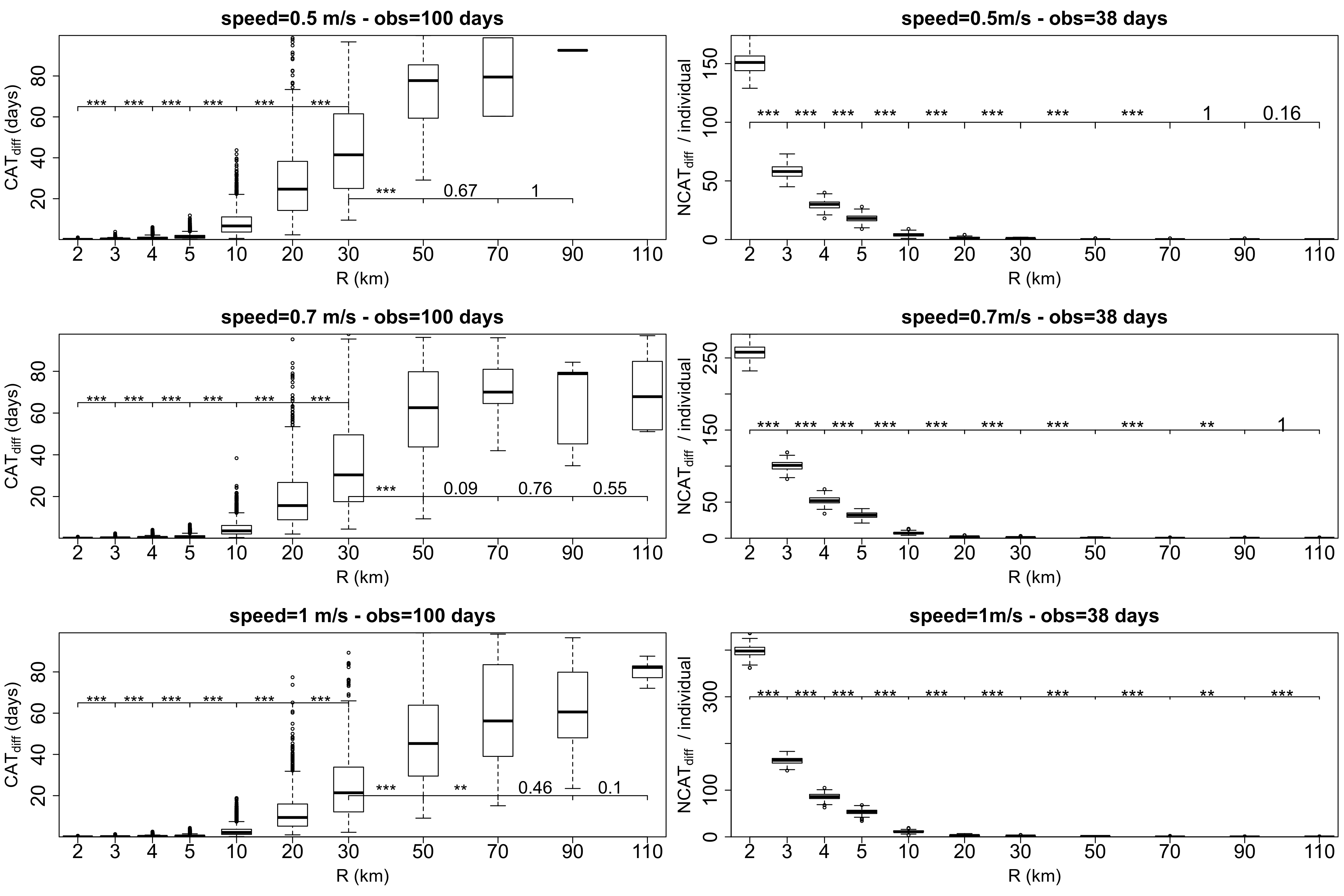

Supplement: Supplementary file 4 — Additional file 4. Random-walk model. [file 40462_2020_230_MOESM4_ESM.zip › AdditionalFile4_S1.png]

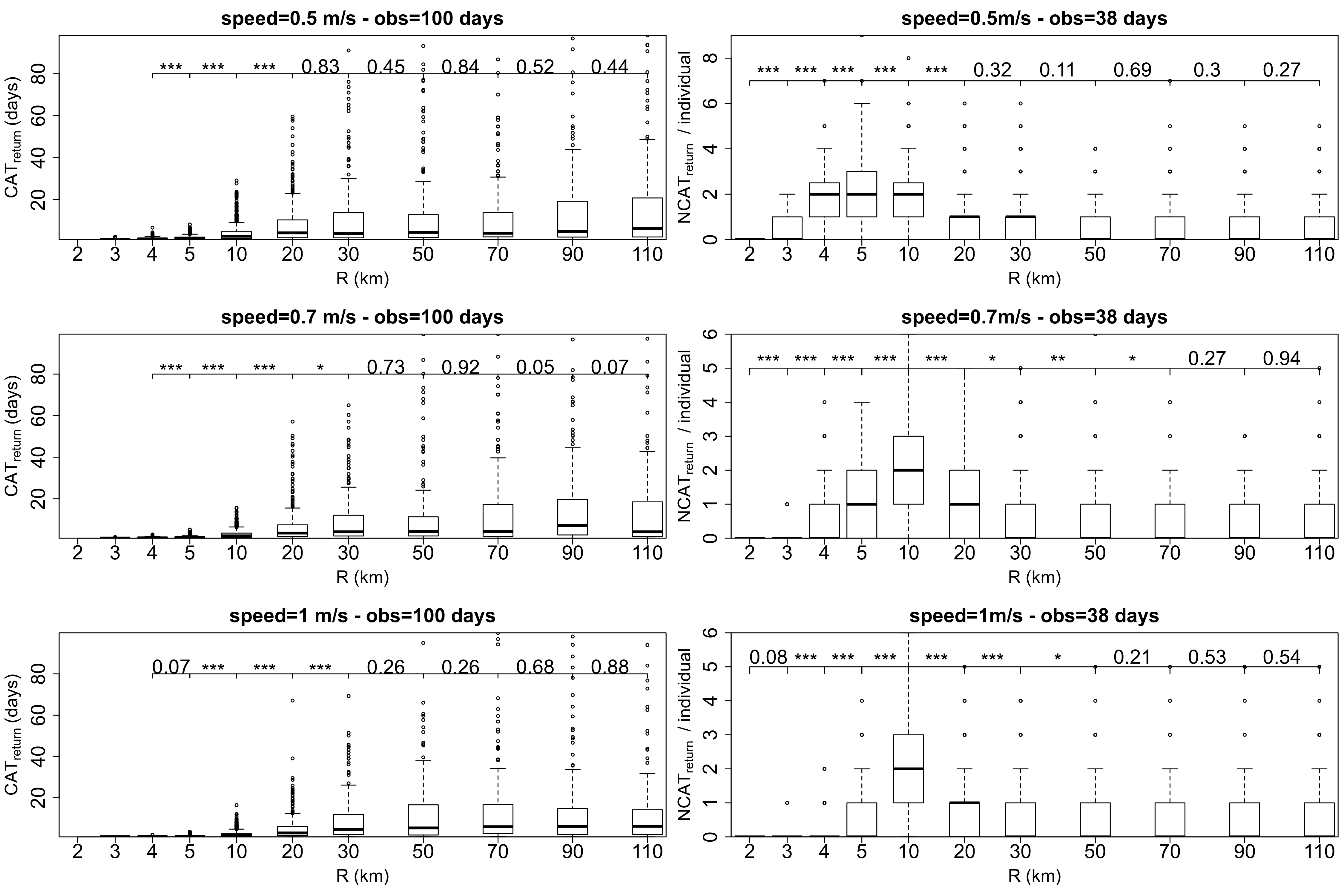

Supplement: Supplementary file 4 — Additional file 4. Random-walk model. [file 40462_2020_230_MOESM4_ESM.zip › AdditionalFile4_S2.png]

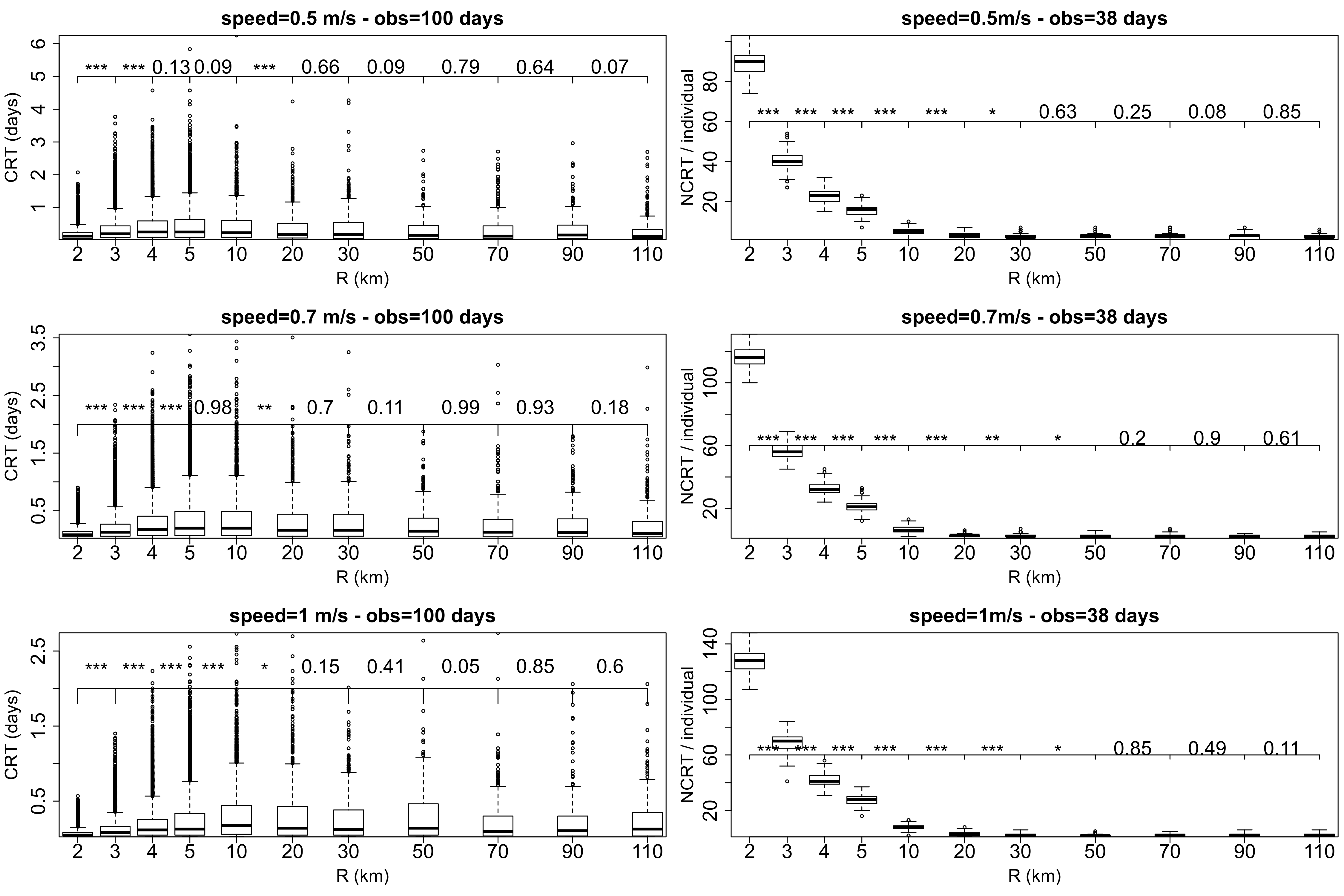

Supplement: Supplementary file 4 — Additional file 4. Random-walk model. [file 40462_2020_230_MOESM4_ESM.zip › AdditionalFile4_S3.png]

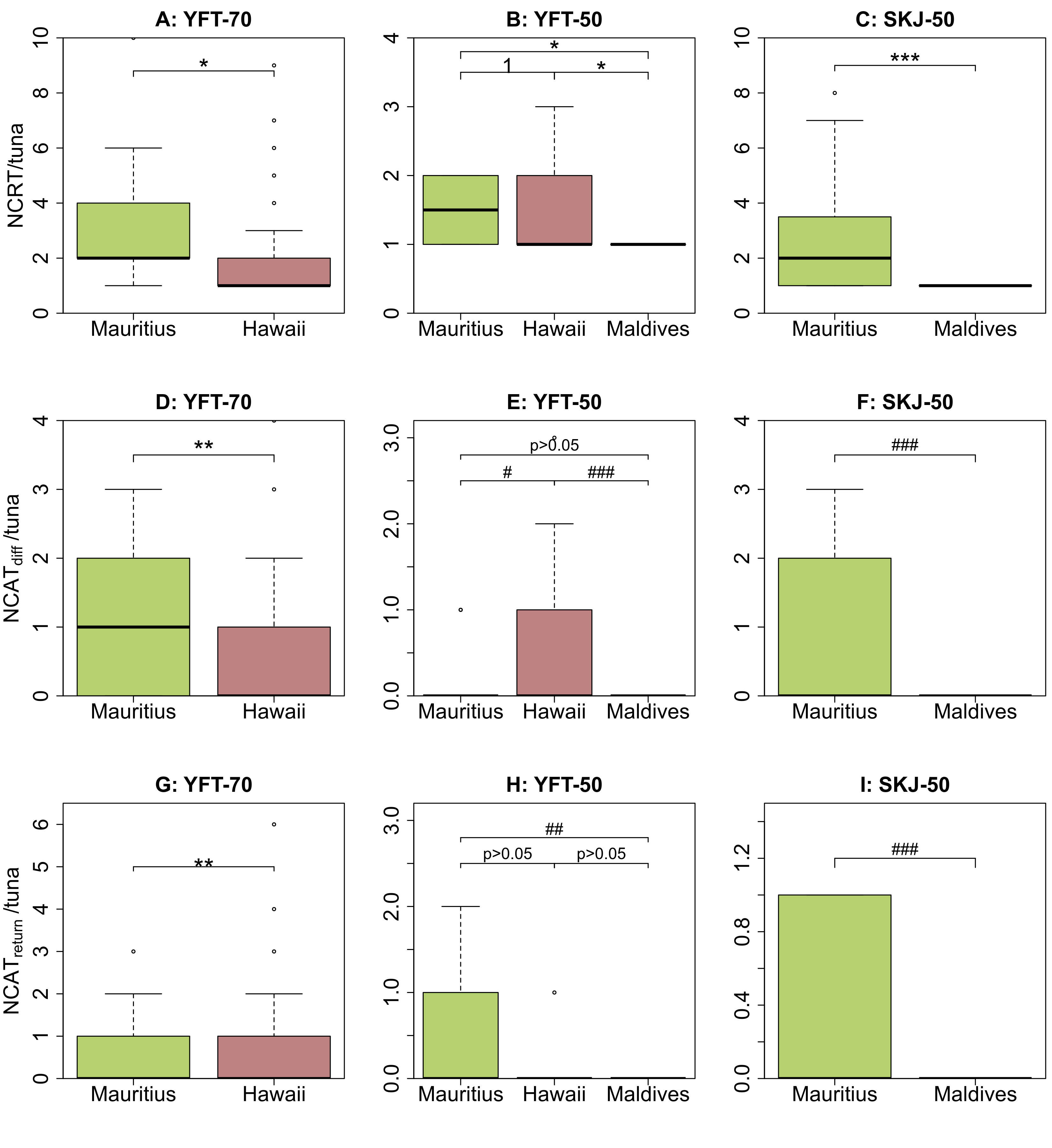

Supplement: Supplementary file 5 — Additional file 5. Number of CRT (NCRT), CATdiff (NCATdiff) and CATreturn (NCATreturn) per individuals for each AFAD array and each specie-size category. YFT-70 (A, D and G), YFT-50 (B, E and H) and SKJ-50 (C, F and I). Mann-Whitney test, except for YFT-50 where a Dunn post hoc test with p-values adjusted by the Holm method was perfornmed: *** indicates p < 0.001, ** p < 0.01, and * p < 0.05. Binomial test: ### indicates p < 0.001, ## p < 0.01, and # p < 0.05. [file 40462_2020_230_MOESM5_ESM.png]
