# Supplementary material for: Effects of habitat modifications on the movement behavior of animals: the case study of Fish Aggregating Devices (FADs) and tropical tunas
Source: Mov Ecol. 2020 Nov 10;8:47. doi: 10.1186/s40462-020-00230-w (PMC7654007; doi:10.1186/s40462-020-00230-w)
Supplement: Supplementary file 2 — Additional file 2. Number of tuna tagged in each FAD array per species and size category. [file 40462_2020_230_MOESM2_ESM.docx]

**Additional file 2**: Number of tuna tagged in each FAD array per species and size category.

| **FAD array** | **Species** | **Size (m)** | **Ntuna** |
| --- | --- | --- | --- |
| **MAURITIUS** | YFT | 0.5 | 11 |
|  |  | 0.7 | 14 |
|  |  | 0.9 | 1 |
|  | BET | 0.5 | 6 |
|  |  | 0.7 | 1 |
|  | SKJ | 0.5 | 15 |
|  | **Total** | | **49** |
| **HAWAII** | YFT | 0.3 | 20 |
|  |  | 0.5 | 9 |
|  |  | 0.7 | 56 |
|  |  | 0.9 | 7 |
|  | **Total** | | **92** |
| **MALDIVES** | YFT | 0.3 | 1 |
|  |  | 0.5 | 19 |
|  | SKJ | 0.3 | 10 |
|  |  | 0.5 | 22 |
|  | **Total** | | **52** |
