# Supplementary material for: Effects of habitat modifications on the movement behavior of animals: the case study of Fish Aggregating Devices (FADs) and tropical tunas
Source: Mov Ecol. 2020 Nov 10;8:47. doi: 10.1186/s40462-020-00230-w (PMC7654007; doi:10.1186/s40462-020-00230-w)
Supplement: Supplementary file 3 — Additional file 3. Number of tuna, number of CRTs, CATreturn(s) and CATdiff(s) for the full observation time and for only the first 38 days of observation. [file 40462_2020_230_MOESM3_ESM.docx]

**Additional file 3**: Number of tuna, number of CRTs, CAT_return_(s) and CAT_diff_(s) for the full observation time and for only the first 38 days of observation.

|  | **YFT 70** | | **YFT 50** | | | | **SKJ 50** | |
| --- | --- | --- | --- | --- | --- | --- | --- | --- |
|  | **Mauritius** | **Hawaii** | **Mauritius** | **Hawaii** | **Maldives** | **Mauritius** | | **Maldives** |
| **Ntuna** | **14** | **56** | **11** | **9** | **19** | **15** | | **22** |
| **FOR ENTIRE MONITORING PERIOD** | | | | | | | | |
| **NCRT** | 43 | 167 | 17 | 25 | 19 | 44 | | 22 |
| **NCAT_return_** | 10 | 51 | 4 | 6 | 0 | 13 | | 0 |
| **NCAT_diff_** | 19 | 60 | 2 | 10 | 0 | 16 | | 0 |
| **FOR THE 38 FIRST DAYS OF OBSERVATION** | | | | | | | | |
| **NCRT** | 42 | 109 | 15 | 15 | 19 | 43 | | 22 |
| **NCAT_return_** | 10 | 25 | 4 | 1 | 0 | 13 | | 0 |
| **NCAT_diff_** | 19 | 39 | 2 | 6 | 0 | 16 | | 0 |
