# Supplementary material for: Effects of habitat modifications on the movement behavior of animals: the case study of Fish Aggregating Devices (FADs) and tropical tunas
Source: Mov Ecol. 2020 Nov 10;8:47. doi: 10.1186/s40462-020-00230-w (PMC7654007; doi:10.1186/s40462-020-00230-w)
Supplement: Supplementary file 4 — Additional file 4. Random-walk model. [file 40462_2020_230_MOESM4_ESM.zip › additionalFile4_trackChange.docx]

**Additional File 4: Random-walk model**

Random-walk models are often used to model animal movements at local and global scales (Sibert et al. 1999; Murray, 2001; Adam & Sibert 2004; Codling et al. 2008; O’Sullivan & Perry, 2013; Ahearn et al. 2017). Most studies that compare the movement patterns of animals with random-walk models are based on animal tracks collected in the field. In the case of passive acoustic telemetry studies of aquatic animal movements, the data take the form of presence/absence of the tagged individuals within the detection range of the acoustic receivers at specific locations (see e.g. Capello et al. 2015)⁠. Subsequent detections at the same receiver constitute the building blocks for assessing the animal’s residence times at a given location whereas detections at different receivers allow the identification of movements, without details on their paths. The patterns of residence times and movements recorded for tagged animals depend on the nature of the animal’s movements but also, by construction, on the distance between the receivers (located on specific locations, e.g. aggregation sites). When considering the effects of increasing the number of aggregation sites on animal movements, it is essential to understand whether the observed changes are due to real changes in the animal behavior due to this habitat change, or to the effects of the modified array of acoustic receivers (installed on the aggregation sites) on the calculated movement indices, even when the animal behavior does not change. To date, no study has compared the behavior of electronically tagged individuals with random walk models using such data. Consequently, simple random-walk simulations are used (1) to evaluate the effects of the distance between FADs (acoustic receivers) on the indices, and (2) to assess whether the observed trends of indices from tagging data could be interpreted in the light of a random-search component in tuna behaviour suggested in previous experiments (Girard et al. 2004).

**1. Random-walk simulations**

We ran simulations from a simple random-walk model in a two-dimensional continuous space.  These models are based on a Markov process, which corresponds to a one-generation time dependency. In other words, the state at time *t* only depends on the state at time *t–Δt,* where Δt is the time step*.* For simple random-walk models defined in a two-dimensional continuous space, a constant speed is assumed. Therefore, the position of an individual depends on its previous position and the turning angle (α), which is randomly drawn from a uniform distribution: α Є [- π,π], defining the change in direction between two consecutive time steps. In this study, simple random-walk simulations were run using three different speeds (0.5 m/s, 0.7 m/s and 1.0 m/s). The two former speed values represented the movements of fish of 50-cm and 70-cm fork length moving at one body-length per second, respectively. The latter speed was considered because tuna can also show swimming speeds larger than one body-length per second (Dagorn et al. 2007). Simulations were performed using a time step of 1000 seconds, which meant that for each time step, an individual travelled 500 m, 700 m and 1 km depending on the speed. This spatial scale was chosen based on the detection range of the acoustic receivers, which, in this type of acoustic telemetry experiment, typically averages at 500 m (Dagorn et al. 2007, Rodriguez-Tress et al. 2017). The simulations took place in a theoretical environment where FADs were regularly spaced in a square lattice of size L. Each simulation lasted 100 days (8640 time steps) and a total of 200 independent individual paths were simulated. All individuals started from a FAD located on the center of the modelled habitat. The value of L was chosen to be large enough (L = 1000 km) to avoid individuals encountering the boundary during the simulated time. A total of 11 different distances between nearest-neighboring FADs were considered: 2, 3, 4, 5, 10, 20, 30, 50, 70, 90 and 110 km. Around each FAD a 500-m radius zone was defined as the detection range of an acoustic receiver, in order to reproduce the manner in which the detection data were actually recorded. Accordingly, each time a walker (simulated individual) was present within this zone, it was considered to be “detected” at a given FAD. When individuals reached a FAD, they directly continued their path and no specific FAD-retention behavior was modelled. Finally, the same data-processing schemes carried out on tagging data to calculate CRT, CAT_diff_ and CAT_return_ were applied on the simulated data. A CRT was therefore calculated the same way it was for actual tagged tuna: from the first to the last “detection” at the FAD (when the walker enters the 500-m radius), as long as the gap between two consecutive detections is less than 24 hours, or that the walker is not “detected” by another FAD. The resulting indices were compared over different FAD array densities using the same methods presented in paragraph 2.3. The simulations were performed using the Python 3 programming language (Python Software Foundation, version 3.7.4).

**2. Results of the random walk model**

**2.1. Absence times**

The random-walk simulations showed that CAT_diff_ increased significantly with increasing inter-FAD distances up to a threshold distance (Mann-Whitney test: p-value<0.05, Figure S1, left column) that increased with the speed. Conversely, the number of CAT_diff_ per simulated individual (NCAT_diff_/individual) decreased significantly with increasing distances (Mann-Whitney test: p-value<0.05, Figure S1 right column) up to larger thresholds. For the speed of 0.5 m/s no CAT_diff_ were recorded, when inter-FAD distances went up to 90 km (Figure S1, right column).

The CAT_return_ increased up until a certain distance between FADs depending on the speed (Mann-Whitney test: p-value<0.05, Figure S2, left column), from which point CAT_return_ did not vary significantly. The number of CAT_return_ per simulated individual (NCAT_return_/individual) showed a non-monotonic trend (Figure S2, right column). Initially, NCAT_return_/individual increased significantly for increasing distances between FADs, then the median value decreased significantly as inter-FAD distances increased further. Finally, for inter-FAD distances larger than a given threshold that depended on the speed, no significant difference was found (Mann-Whitney test: p-value>0.05 Figure S2 right column).

**Figure S**1: Trends of CAT_diff_ and number of CAT_diff_ per simulated individual (NCAT_diff_/individual) for increasing inter-FAD distances and different values of the speed.

The CAT_diff_ (left column) and NCAT_diff_/individual (right column) are showed for each inter-FAD distance. Each row corresponds to a different swimming speed. Mann-Whitney test: *** indicates p<0.001, ** p<0.01, and * p<0.05.

**Figure S**2: Trends of CAT_return_ and number of CAT_return_ per simulated individual (NCAT_return_/individual) for increasing inter-FAD distances and different values of the speed.

The CAT_return_ (left column) and NCAT_return_/individual (right column) are showed for each inter-FAD distance. Each row corresponds to a different swimming speed. Mann-Whitney test: *** indicates p<0.001, ** p<0.01, and * p<0.05.

**2.2. Residence times**

The random-walk simulations showed that CRT increased significantly when the inter-FAD distances increased between FADs (Mann-Whitney test: p-value<0.05, Figure S3 left column). On the contrary, the number of CRTs per simulated individual decreased significantly with the inter-FAD distance (Mann-Whitney test: p-value<0.05 Figure S3 left column). The two indices stabilized at a given threshold that depended on the swimming speed, beyond which they did not differ significantly (Mann-Whitney test: p-value>0.05, Figure S3).

**Figure S**3: Trends of CRT and NCRT per simulated individual (NCRT/individual) for increasing inter-FAD distances and different values of the speed.

The CRT (left column) and NCRT/individual (right column) are showed for each inter-FAD distance. Each row corresponds to a different swimming speed. Mann-Whitney test: *** indicates p<0.001, ** p<0.01, and * p<0.05.

**3. Discussion of the results obtained for the random walk simulations**

Since the simple random-walk model has no particular FAD associative behaviour (no FAD-attraction nor FAD-retention terms), obviously, the walker’s dynamics does not change when distances between FADs change. Therefore, the trends observed on the indices when inter-FAD distances increased were only due to the effects of the spatial design of the array of receivers.

For a random walker, CAT_diff_(s) logically increase (and become fewer) when inter-FAD distances increase, as it takes longer to move a greater distance when speed is constant. Furthermore, beyond a threshold distance between FADs (receivers), under the duration of the simulation and the speed value considered, CAT_diff_(s) become too long to be recorded (see Figure S1, left column). After leaving a particular FAD, the random walker can also randomly return to it, and by definition, if the excursion lasts more than 24 hours, resulting in a CAT_return_. The results of the simulations show that CAT_return_(s) increase when inter-FAD distances increase, as there is a decreased likelihood of randomly finding another FAD. Since the simulations have a finite duration, as do the experiments, this increase reaches a plateau such that longer CAT_return_(s) cannot be recorded (Figure S2, left column). The number of CAT_diff_ per simulated individual was highest at the smallest inter-FAD distance, decreased with increasing distances and remained constant when inter-FAD distances became large (Figure S1, right column). Clearly, when FADs (receivers) are very close to each other, each time the random walker leaves a FAD, it is likely to be detected by another receiver, thus showing very few CAT_return_(s) (Figure S2 right column). As the inter-FAD distance increases, once receivers are far enough apart to allow returns to the same FAD to occur, the random walker can perform one or multiple CAT_return_(s) for any FAD visited. However, above a certain distance between FADs (receivers), the random walker will encounter fewer FADs, and CAT_return_(s) will logically become longer in duration and fewer in number. This explains the non-monotonous trend recorded in the NCAT_return_ for increasing inter-FAD distances. The plateau observed for the number and the duration of CAT_return_, even if the distances between FADs (receivers) increase, is due to the finite duration of the simulations, which sets the maximum duration and number of CAT_return_. Finally, for residence times, the random**-**walk simulations show longer CRTs with increasing inter-FAD distances until this duration reaches a plateau (Figure S3, left column). By definition, if an excursion away from a FAD lasts less than 24 hours and the random walker does not encounter another FAD, the CRT is not interrupted. If FADs are very close to each other, this implies that the walker performing a short excursion (< 24 h) could randomly encounter another FAD, which would interrupt the CRT and start a new one. This increase in the duration of CRT with the inter-FAD distances occurs until a certain threshold that corresponds to the maximum distance that the walker can travel in 24 hours (by construction, the maximum time window allowed between two consecutive detections at the same receiver to consider that a fish is still associated) (Ohta and Kakuma 2005, Capello et al. 2015). In summary, for inter-FAD distances larger than a given threshold, which depends on an individual’s speed, the walkers no longer encounter other receivers during their short excursions (< 24h) and thus the duration of CRTs reaches a plateau.

**4. References**

Adam S. M., and Sibert J. R. (2002). Population Dynamics and Movements of Skipjack Tuna (Katsuwonus Pelamis) in the Maldivian Fishery: Analysis of Tagging Data from an Advection-Diffusion-Reaction  Model. *Aquatic Living Resources* 15: 13–23.

Ahearn, S. C., Dodge, S., Simcharoen, A., Xavier, G., & Smith, J. L. D. (2017). A context-sensitive correlated random walk: a new simulation model for movement. *International Journal of Geographical Information Science*, *31*(5), 867–883.

Capello, M., Robert, M., Soria, M., Potin, G., Itano, D., Holland, K., J-L Deneubourg, Dagorn, L. (2015). A methodological framework to estimate the site fidelity of tagged animals using passive acoustic telemetry. *PLoS ONE*, *10*(8), 1–19.

Codling, E. A., Plank, M. J., & Benhamou, S. (2008). Random walk models in biology. *Journal of the Royal Society Interface*, *5*(25), 813–834. https://doi.org/10.1098/rsif.2008.0014

Dagorn, L., Holland, K. N., & Itano, D. G. (2007). Behavior of yellowfin (Thunnus albacares) and bigeye (T. obesus) tuna in a network of fish aggregating devices (FADs). *Marine Biology*, *151*(2), 595–606. https://doi.org/10.1007/s00227-006-0511-1

Murray, J. D. (2001). Mathematical Biology: I. An Introduction, Third Edition (Spinger).

Ohta, I., & Kakuma, S. (2005). Periodic behavior and residence time of yellowfin and bigeye tuna associated with fish aggregating devices around Okinawa Islands, as identified with automated listening stations. *Marine Biology*, *146*(3), 581–594. https://doi.org/10.1007/s00227-004-1456-x

O’Sullivan, D., & Perry, G. (2013). Spatial Simulation: Exploring Pattern and Process. (Wiley-Blackwell, Ed*)*.

Sibert, J., Hampton, J., Fournier, D.A., and Bills, P.J. (1999). An Advection-Diffusion-Reaction Model for the Estimation of Fish Movement Parameters from Tagging Data, with Application to Skipjack Tuna (Katsuwonus Pelamis). *Canadian Journal of Fisheries and Aquatic Sciences* 56, no. 6: 925–38.
